# Overexpression of miR-29a reduces the oncogenic properties of glioblastoma stem cells by downregulating Quaking gene isoform 6

**DOI:** 10.18632/oncotarget.15327

**Published:** 2017-02-15

**Authors:** Zhuo Xi, Ping Wang, Yixue Xue, Chao Shang, Xiaobai Liu, Jun Ma, Zhiqing Li, Zhen Li, Min Bao, Yunhui Liu

**Affiliations:** ^1^ Department of Neurosurgery, Shengjing Hospital of China Medical University, Shenyang 110004, People's Republic of China; ^2^ Liaoning Research Center for Translational Medicine in Nervous System Disease, Shenyang 110004, People's Republic of China; ^3^ Department of Neurobiology, College of Basic Medicine, China Medical University, Shenyang 110122, People's Republic of China

**Keywords:** miR-29a, QKI-6, WTAP, glioma, glioblastoma stem cells

## Abstract

Glioblastoma is the most common type of malignant primary brain tumor and has high recurrence and lethality rates. Glioblastoma stem cells (GSCs), a subpopulation of glioblastoma cells, may promote rapid tumor recurrence and therapy resistance. Because altered microRNA (miR) expression in GSCs may lead to glioblastoma progression, we assessed the effects of miR-29a expression on the oncogenic behavior of GSCs. MiR-29a expression was lower in GSCs than non-GSCs, and overexpression of miR-29a in GSCs inhibited cell proliferation, migration and invasion, but promoted apoptosis. MiR-29a directly inhibited the expression of Quaking gene isoform 6 (*QKI-6*) by binding to its 3′-UTR, and thus inhibited GSC malignant behavior. In addition, Wilms’ tumor 1-associating protein (*WTAP*) was identified as a downstream target of QKI-6. Overexpression of miR-29a in GSCs inhibited expression of WTAP and suppressed both phosphoinositide 3-kinase/AKT and extracellular signal-related kinase pathways by downregulating QKI-6, thereby inhibiting cell proliferation, migration, and invasion but promoting apoptosis. We have characterized a novel miR-29a/QKI-6/WTAP axis in GSCs, which may provide theoretical support for the treatment of glioblastoma with miR-29a agomirs.

## INTRODUCTION

Glioblastoma, regarded as a grade IV astrocytoma, is the most common and malignant type of primary brain tumor. Some studies have demonstrated that glioblastomas contain a small subpopulation of cells with the ability to self-renew and to initiate brain tumors. These cells are considered to be glioblastoma stem cells (GSCs), which promote tumor progression, treatment resistance, and tumor recurrence [1–3]. Novel therapies focusing on GSCs, including molecular targeted therapies, immunotherapies and gene therapies, have emerged for glioblastoma treatment.

MicroRNAs (miRNAs) are small, non-coding RNA molecules of approximately 19-22 nucleotides. Many recent studies have revealed that miRNAs regulate the behavior of GSCs and may have similar functions to oncogenes or tumor suppressors [4]. For example, miR-20a and miR-106a enhance the invasiveness of GSCs [5], whereas miR-145 inhibits the migration and invasion of GSCs [6]. MiR-29a belongs to the miR-29 family, which consists of miR-29a, -29b and -29c. The structure, function, and regulation of miR-29s are highly conserved in humans, mice and rats. MiR-29a has been found to be downregulated in lung cancer and invasive pancreatic cancer [7], but upregulated in breast cancer [8]. While miR-29a expression has also been reported to be reduced in gliomas [9], the molecular pathways regulated by miR-29a and their influence on GSC biological processes are not yet known.

The *Quaking (QKI)* gene was initially defined for its role in myelination in the central nervous system. Three major alternatively spliced mRNAs of *QKI* (5, 6, and 7kb) are expressed, encoding the proteins QKI-5, QKI-6 and QKI-7, which differ only in their 30 C-terminal amino acids. The QKI proteins selectively bind to a short sequence, the QKI response element (QRE), which is frequently located in the 3′-untranslated regions (UTRs) of target mRNAs [10]. The QKI isoforms play a functional role in pre-mRNA splicing, mRNA stabilization, protein translation and other cellular processes during glial, oligodendrocyte and Schwann cell differentiation, in both the central and peripheral nervous systems [11–13]. In the present study, the potential functions of QKI-6 in glioblastoma and GSCs are discussed.

Wilms’ tumor 1-associating protein (WTAP) binds to the Wilms’ tumor 1 gene (*WT1*). Although originally classified as a tumor suppressor in nephroblastoma [14], WT1 was later found to be overexpressed in many types of cancer, including breast carcinoma, acute leukemia and glioblastoma [15–17]. Because binding to WT1, WTAP takes part in RNA splicing and stabilization. Jin et al. initially found that WTAP was overexpressed in glioblastomas, and that it promoted the migration and invasion of glioblastoma cells by stimulating epidermal growth factor (EGF) signaling [18]. Whether WTAP participates in the biological processes of miR-29a in GSCs remains unknown.

In this study, we provide evidence that miR-29a is a tumor suppressor in GSCs. Overexpression of miR-29a impaired the oncogenic abilities of GSCs by downregulating QKI-6, ultimately downregulating WTAP and inactivating the phosphoinositide 3-kinase (PI3K)/AKT and extracellular signal-related kinase (ERK) pathways. These results have promising applications for agomir-based glioblastoma treatment.

## RESULTS

### MiR-29a expression is downregulated in GSCs

**Figure 1:**
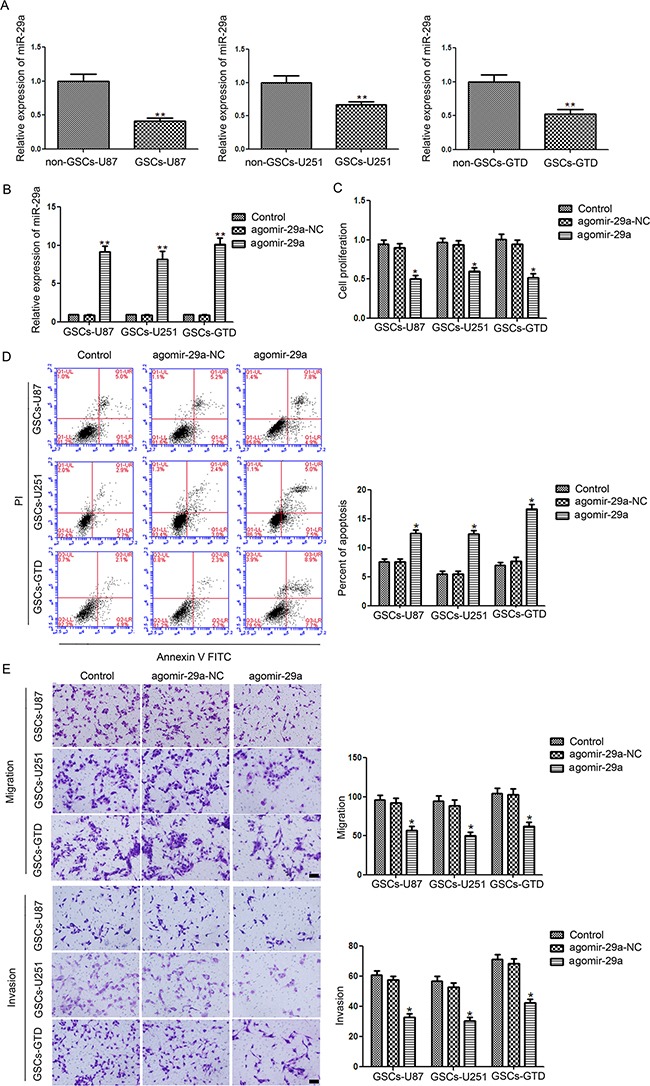
Expression and function of miR-29a in GSCs **A**. Relative expression of miR-29a in GSCs (CD133+) and non-GSCs (CD133-) isolated from U87 and U251 cell lines and glioblastoma tissues. ^*^P<0.01 vs. non-GSC group. **B**. Relative expression of miR-29a after transfection of GSCs with agomir-29a or agomir-29a-NC which were tested by Real-time PCR. ^*^P<0.01 vs. agomir-29a-NC group. **C**. CCK8 assay to evaluate the effect of miR-29a on GSC proliferation **D**. Flow cytometry analysis of GSCs overexpressing miR-29a. **E**. Quantification of cell migration and invasion in GSCs overexpressing miR-29a. Representative images and accompanying statistical plots are presented. For panels C, D, and E, *P<0.05 vs. agomir-29a-NC group. All values represent the mean ± SD from five independent experiments. Scale bar represents 80 μm. The photographs were taken at 200× magnification.

Real-time PCR analysis revealed that miR-29a expression was significantly lower in U87-, U251-, and glioblastoma tissue-derived GSCs (henceforward called GSCs-U87, GSCs-U251 and GSCs-GTD, respectively) than in their respective non-GSCs (all *P*<0.01, Figure 1A).

### Overexpression of miR-29a in GSCs inhibits cell proliferation, migration and invasion but promotes apoptosis

We then overexpressed miR-29a in GSCs-U87, GSCs-U251 and GSCs-GTD with a specific agomir (agomir-29a). The transfection efficiency of miR-29a was nearly 10 times higher in agomir-29a groups than that in the NC groups (all *P*<0.05, Figure 1B). As shown in Figure 1C, the proliferation of GSCs was lower in the agomir-29a groups than in the respective agomir-29a-NC groups (all *P*<0.05), while there were no significant difference between the non-GSC control groups and the agomir-29a-NC groups. The rates of GSC-U87 apoptosis in the agomir-29a and agomir-29a-NC groups were 12.7% and 7.4%, respectively, corresponding to a significant 5.3% increase in the agomir-29a group (*P*<0.05, Figure 1D). In GSCs-U251 and GSCs-GTD, the apoptosis rates were significantly increased by 7.1% (*P*<0.05) and 8.6% (*P*<0.05), respectively, in the agomir-29a groups compared with the NC groups (Figure 1D). The migration and invasion of GSCs-U87, GSCs-U251 and GSCs-GTD were lower in their respective agomir-29a groups than in their agomir-29a-NC groups (all *P*<0.05) (Figure 1E).

### QKI-6 is upregulated in glioblastoma tissues and GSCs

**Figure 2:**
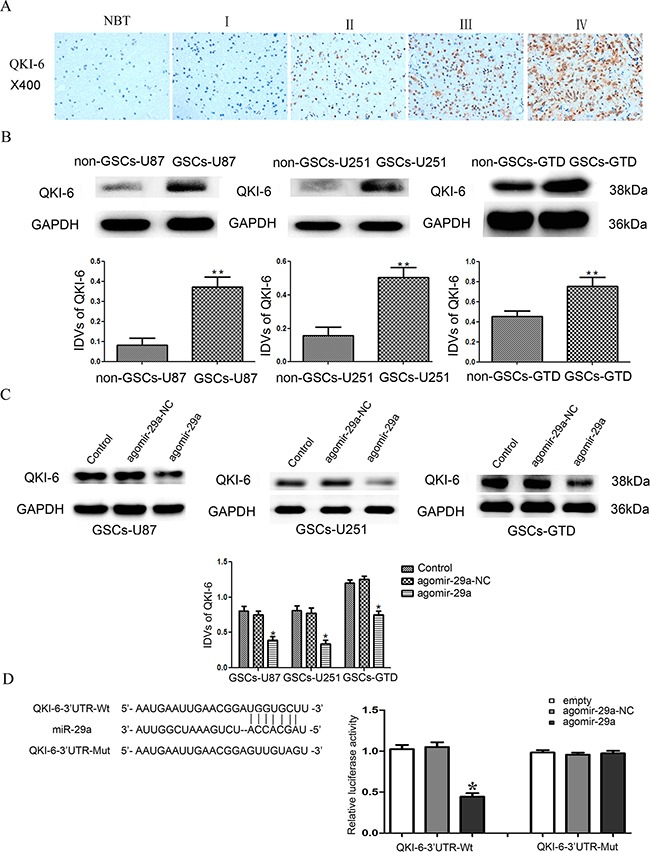
*QKI-6* is a direct target of miR-29a **A**. Representative IHC assay patterns of QKI-6 expression in glioma tissues and normal brain tissues on tissue microarray sections. The photographs were taken at 400× magnification. **B**. Expression of QKI-6 in GSCs (CD133+) and non-GSCs (CD133-), with GAPDH as the endogenous control. ^*^P<0.01 vs. non-GSC group. **C**. Overexpression of miR-29a inhibited QKI-6 expression in GSCs; GAPDH was used as the endogenous control. *P<0.05 vs. agomir-29a-NC group. **D**. Reporter vector constructs and luciferase assays. *P<0.05 vs. *QKI-6*-3′-UTR-Wt+agomir-29a-NC group. IDVs represent the relative integrated density values. For B-D, values represent the mean ± SD from five independent experiments.

We next performed a bioinformatic analysis, which identified *QKI-6* as a predicted target of miR-29a. Thus, immunohistochemistry (IHC) and Western blot analysis were used to evaluate the expression of QKI-6 in glioma tissues. The IHC assay results indicated that QKI-6 is localized mainly in the nucleus and partly in the cytoplasm of glioma cells (Figure 2A). QKI-6 staining correlated positively with the World Health Organization (WHO) grade (r=0.277, *P*<0.05, Table 1). In addition, Western blot analysis demonstrated that the expression of QKI-6 was significantly greater in GSCs-U87, GSCs-U251 and GSCs-GTD than in their respective non-GSCs (all *P*<0.01) (Figure 2B).

**Table 1:** Association of QKI-6 and WTAP with WHO grade

	Number of patients	QKI-6 staining				r	P value	WTAP staining				r	P value
		−	+	++	+++			−	+	++	+++		
Total	60	6	1	30	23			3	4	23	30		
Normal tissues	3	1	0	2	0			2	1	0	0		
WHO grade						0.277	0.032					0.328	0.011
I	3	2	0	1	0			0	0	3	0		
II	9	0	0	8	1			0	2	3	4		
III	11	0	0	5	6			0	0	5	6		
IV	34	3	1	14	16			1	1	12	20		

P value was estimated by Spearman's correlation test

### MiR-29a inhibits QKI-6 expression by binding to the *QKI-6* 3′-UTR

Western blot results indicated that miR-29a overexpression significantly reduced the protein levels of QKI-6 in GSCs-U87, GSCs-U251 and GSCs-GTD compared with their respective NC groups (all *P*<0.05). Thus, the protein expression of QKI-6 in GSCs was inversely related to the expression of miR-29a, suggesting that *QKI-6* might be a target of miR-29a (Figure 2C). To determine whether the *QKI-6*-3′-UTR is a direct target of miR-29a, we co-transfected HEK293T cells with agomir-29a and a *QKI-6*-3′-UTR reporter construct (*QKI-6*-3′-UTR-Wt), and performed luciferase activity assays. Overexpression of miR-29a significantly reduced the luciferase activity of *QKI-6*-3′-UTR-Wt (*P*<0.05), while transfection with agomir-29a-NC did not. These results indicated that miR-29a targets the 3′-UTR of *QKI-6*. To determine whether miR-29a directly binds to *QKI-6* at its putative binding site, we co-transfected HEK293T cells with agomir-29a and a mutated *QKI-6*-3′-UTR reporter construct (*QKI-6*-3′-UTR-Mut). In these experiments, overexpression of miR-29a did not reduce the luciferase activity, indicating that miR-29a directly binds to *QKI-6* at the predicted specific binding site (Figure 2D).

### QKI-6 is oncogenic in human GSCs

**Figure 3:**
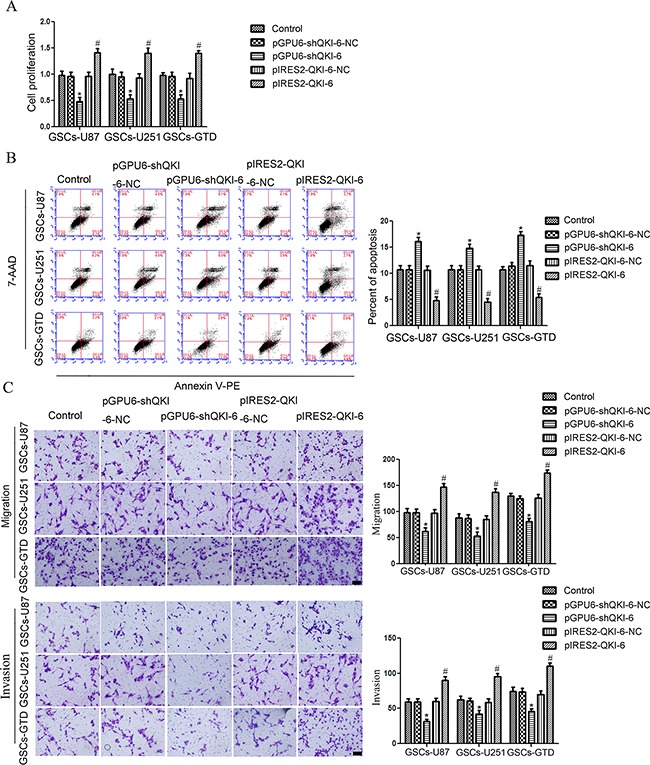
QKI-6 is oncogenic in GSCs **A**. CCK8 assay to evaluate the effect of QKI-6 on GSC proliferation. **B**. Flow cytometry analysis of GSCs with altered QKI-6 expression. **C**. Quantification of cell migration and invasion in GSCs with altered QKI-6 expression. Representative images and accompanying statistical plots are presented. Values represent the mean ± SD from five independent experiments. *P<0.05 vs. pGPU6-shQKI-6-NC group, ^#^*P*<0.05 vs. pIRES2-QKI-6-NC group. Scale bar represents 80 μm. The photographs were taken at 200× magnification.

GSCs-U87, -251 and -GTD were then transfected with plasmids to overexpress *QKI-6* (pIRES2-QKI-6) or knock down QKI-6 (pGPU6-shQKI-6), or with the respective negative control plasmids (pIRES2-QKI-6-NC and pGPU6-shQKI-6-NC). GSC proliferation was greater in the *QKI-6*-overexpressing groups than in the corresponding NC groups, whereas it was lower in the QKI-6-knockdown groups than in the NC groups (all *P*<0.05, Figure 3A). Similarly, GSC-U87 apoptosis was 5.5% lower in the *QKI-6*-overexpressing group (4.9%) than in the NC group (10.4%) (*P*<0.05), whereas the apoptotic rate was 4.7% greater in the QKI-6-knockdown group (15.4%) than in the NC group (10.7%) (*P*<0.05) (Figure 3B). The apoptotic rates in GSCs-U251 and GSCs-GTD followed the same tendency as seen in the GSCs-U87. The migration and invasion of GSCs were significantly greater in the *QKI-6*-overexpressing groups than in the respective NC groups, but were significantly lower in the QKI-6-knockdown groups than in the respective NC groups (all *P*<0.05) (Figure 3C).

### Overexpression of miR-29a in GSCs inhibits cell proliferation, migration and invasion but promotes apoptosis by downregulating QKI-6

**Figure 4:**
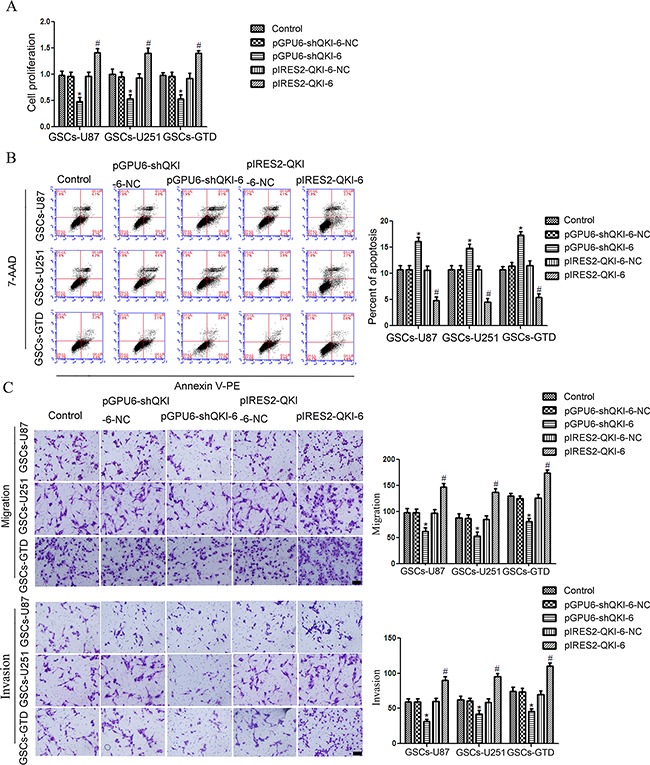
Overexpression of miR-29a inhibits GSC oncogenesis by downregulating QKI-6 **A**. Overexpression of miR-29a inhibited GSC proliferation by downregulating QKI-6. **B**. Overexpression of miR-29a combined with QKI-6 inhibition led to the highest apoptosis rate in GSCs. **C**. Overexpression of *QKI-6* reversed the inhibitory effects of miR-29a overexpression on GSC migration and invasion. Representative images and accompanying statistical plots are presented. Values represent the mean ± SD from five independent experiments.*P<0.05 vs. agomir-29a-NC group,^#^*P*<0.05 vs. agomir-29a-NC+pGPU6-shQKI-6-NC group, ^&^*P*<0.05 vs. agomir-29a group. Scale bar represents 80 μm. The photographs were taken at 200× magnification.

To determine whether QKI-6 could counteract the effects of miR-29a on cell proliferation, migration, invasion and apoptosis (see Figure 1), we simultaneously overexpressed miR-29a and QKI-6 in GSCs by co-transfecting the cells with agomir-29a and pIRES2-QKI-6. Overexpression of QKI-6 reversed the inhibitory effects of miR-29a overexpression on GSCs-U87, GSCs-U251 and GSCs-GTD proliferation (Figure 4A). Thus, the proliferation of GSCs co-transfected with agomir-29a and pIRES2-QKI-6 did not differ significantly from that of the respective NC groups or the control groups. On the other hand, GSC proliferation was lower in both the agomir-29a groups and the agomir-29a+pGPU6-shQKI-6 groups than in the separate NC groups. Furthermore, proliferation was significantly lower in the agomir-29a+pGPU6-shQKI-6 groups than in the agomir-29a groups (all *P*<0.05). There were no clear differences between the control groups and the NC groups.

As shown in Figure 4B, the apoptosis rates of GSCs-U87, GSCs-U251 and GSCs-GTD in the agomir-29a+pIRES2-QKI-6 groups were significantly lower than those in the agomir-29a groups (all *P*<0.05), but did not differ significantly from those of the respective NC groups. The apoptosis rates in the agomir-29a and agomir-29a+pGPU6-shQKI-6 groups were both greater than those of the respective NC groups. The apoptosis rates were also significantly greater in the agomir-29a+pGPU6-shQKI-6 groups than in the agomir-29a groups, demonstrating that QKI-6 inhibition induces the apoptosis of GSCs, just as miR-29a overexpression does (all *P*<0.05). There were no clear differences between the control groups and the NC groups.

As shown in Figure 4C, the migration and invasion of GSCs followed the same trend as proliferation - overexpression of *QKI-6* reversed the inhibitory effects of miR-29a overexpression on GSC migration and invasion (all *P*<0.05).

### MiR-29a overexpression combined with QKI-6 knockdown suppresses tumor growth and increases the survival rate of nude mice

As shown in Supplementary Figure 1A, GSCs overexpressing miR-29a, pGPU6-shQKI-6, or both were injected subcutaneously into the right axillary fossa of nude mice. Tumor size was measured every five days until 60 days after injection, then we resected the tumors and put them together under the same measuring scale (Supplementary Figure 1B). The tumors were smaller in the miR-29a overexpression group and the QKI-6 inhibition group than in their respective control groups (Supplementary Figure 1C). The smallest tumors were observed when miR-29a overexpression was combined with QKI-6 inhibition.

As shown in Supplementary Figure 1D, GSCs overexpressing miR-29a, pGPU6-shQKI-6, or both were then injected into the right striatum of nude mice. Survival analysis revealed that miR-29a overexpression combined with QKI-6 inhibition resulted in the greatest survival rate among the treatment groups. The miR-29a overexpression group and the QKI-6 inhibition group had greater survival rates than their respective control groups.

### *WTAP* is a direct downstream target of QKI-6

**Figure 5:**
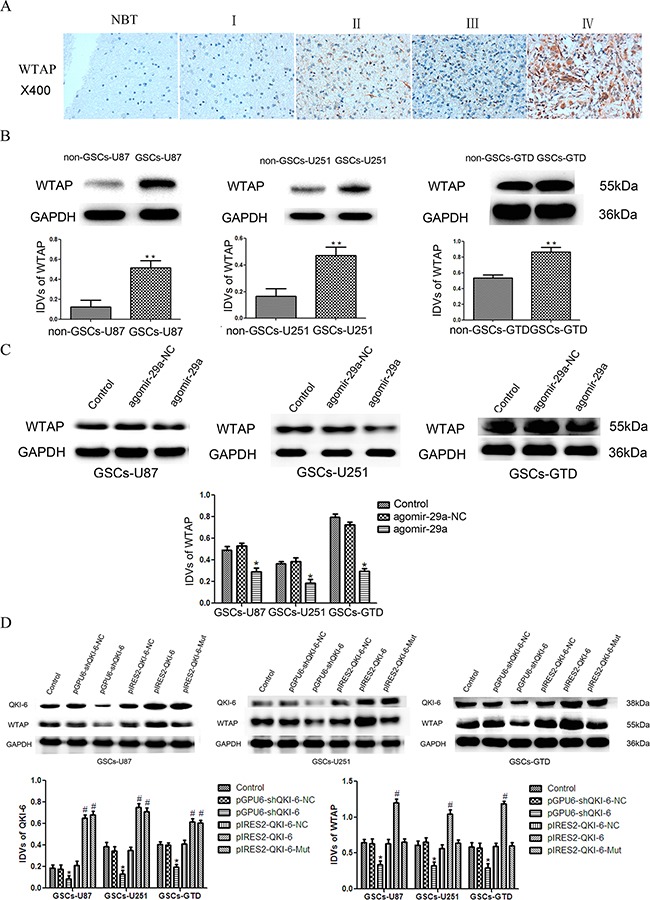
*WTAP* is a direct target of QKI-6 **A**. IHC assay images of WTAP expression in glioma tissues and normal brain tissues on tissue microarray sections. The photographs were taken at 40× magnification. **B**. WTAP expression was greater in GSCs (CD133+) than in non-GSCs (CD133-) in Western blot analysis with GAPDH as an endogenous control. ^*^P<0.01 vs. non-GSC group. **C**. WTAP expression was significantly lower in miR-29a-overexpressing GSCs-U87, GSCs-U251 and GSCs-GTD than in their respective NC groups. *P<0.05 vs. agomir-29a-NC group. **D**. The protein expression of WTAP in GSCs after *QKI-6* overexpression and inhibition. *P<0.05 vs. pGPU6-shQKI-6-NC group,^#^*P*<0.05 vs. pIRES2-QKI-6-NC group. For B-D, values represent the mean ± SD from five independent experiments. IDVs represent the relative integrated density values.

We used bioinformatics databases (Starbase v2.0, miRcode and RNAhybrid) to search for downstream targets of QKI-6. *WTAP* was not predicted to harbor any putative miR-29a binding sites in its 3′-UTR, suggesting that it is not regulated directly by miR-29a. Rather, it seemed probable that *WTAP* is a target of QKI-6, because *WTAP* contains the QRE [ACCAAC-(N_3_)-CAAU] motif in its 3′-UTR. The QRE includes a core ACCAAC sequence and a half-site CAAU, to which QKI-6 may bind and thus regulate *WTAP* expression. To determine whether WTAP participates in the tumor suppression pathway of miR-29a in GSCs, we evaluated WTAP expression in glioma tissues and GSCs. An IHC assay on tissue microarray chips (containing the same tissue samples used in the QKI-6 IHC assay) indicated that WTAP is localized throughout the nucleoplasm in glioma cells (Figure 5A) and is overexpressed in glioma tissues (especially glioblastoma tissues) compared with normal brain tissues. WTAP staining correlated positively with the WHO grade (r=0.328, *P*<0.05, Table 1). Additionally, WTAP expression was significantly greater in GSCs-U87, GSCs-U251 and GSCs-GTD than in their respective non-GSCs in Western blot analysis (all *P*<0.01, Figure 5B).

Overexpression of miR-29a significantly reduced WTAP expression in GSCs-U87, GSCs-U251 and GSCs-GTD, compared with their respective NCs (all *P*<0.05, Figure 5C). To determine whether QKI-6 could regulate the expression of *WTAP* directly, we used plasmid vectors to either knock down QKI-6, overexpress wild-type *QKI-6* (pIRES2-QKI-6), or overexpress a mutant form of *QKI-6* missing the *WTAP* binding sites (pIRES2-QKI-6-Mut). WTAP expression was lower in QKI-6-knockdown GSCs-U87, GSCs-U251 and GSCs-GTD than in their respective NC groups (all *P*<0.05, Figure 5D). WTAP expression was greater in the *QKI-6*-overexpressing GSCs than in the respective NC groups (all *P*<0.05), and was much greater in the pIRES2-QKI-6 groups than in the pIRES2-QKI-6-Mut groups, but did not differ significantly between the *QKI-6* mutant groups and the NC groups. These results demonstrate that QKI-6 can directly regulate *WTAP* expression.

### Overexpression of miR-29a inhibits WTAP expression and the activation of the ERK and PI3K/AKT pathways by downregulating QKI-6

**Figure 6:**
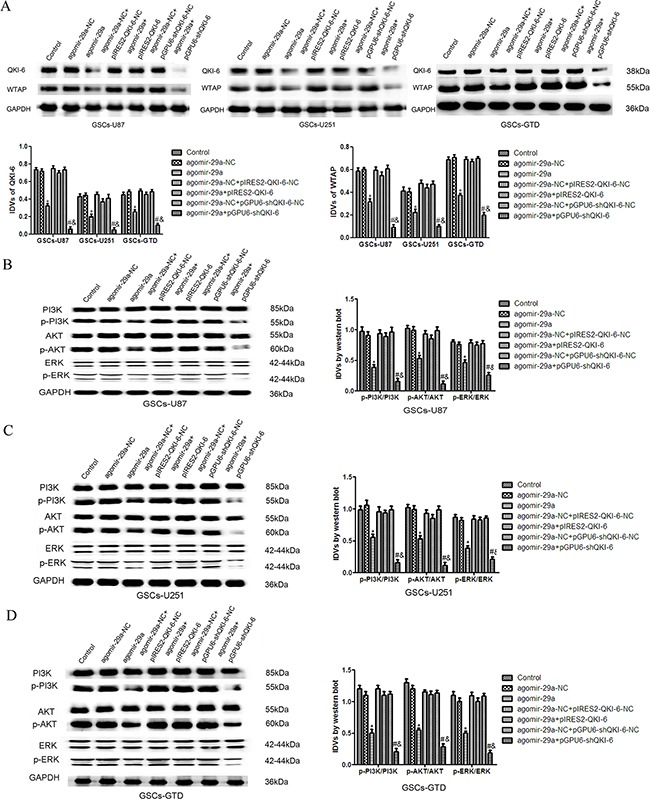
Overexpression of miR-29a inhibits the expression of WTAP and activation of the PI3K/AKT/ERK pathways by downregulating QKI-6 **A**. Western blot analysis of WTAP expression, as regulated by miR-29a and QKI-6 in GSCs. **B**. The expression of p-PI3K/PI3K, p-AKT/AKT and p-ERK/ERK in GSCs-U87 with altered expression of miR-29a and QKI-6. **C**. Western blot analysis of p-PI3K/PI3K, p-AKT/AKT and p-ERK/ERK expression, as regulated by miR-29a and QKI-6 in GSCs-U251. **D**. Overexpression of miR-29a inhibited the activation of the p-PI3K/PI3K, p-AKT/AKT and p-ERK/ERK pathways by downregulating QKI-6 in GSCs-GTD. GAPDH was used as an internal loading control. Accompanying graphs display the densitometry analysis of protein expression. Values represent the mean ± SD from five independent experiments.*P<0.05 vs. agomir-29a-NC group,^#^*P*<0.05 vs. agomir-29a-NC+pGPU6-shQKI-6-NC group, ^&^*P*<0.05 vs. agomir-29a group. IDVs represent the relative integrated density values.

To determine whether miR-29a inhibits the expression of WTAP by downregulating *QKI-6*, we transfected GSCs-U87, GSCs-U251 and GSCs-GTD with a variety of plasmid combinations. As shown in Figure 6A, the expression of WTAP in the agomir-29a+pIRES2-QKI-6 groups was higher than that in the respective agomir-29a groups and did not differ significantly different from that in the NC groups, indicating that the overexpression of *QKI-6* reversed the inhibitory effect of miR-29a on WTAP expression. Conversely, WTAP expression was significantly lower in GSCs treated with agomir-29a+pGPU6-shQKI-6 than in those treated with the NC (all *P*<0.05). Furthermore, miR-29a overexpression combined with QKI-6 inhibition reduced WTAP expression to a greater extent than miR-29a overexpression alone (all *P*<0.05). There were no significant differences between the control groups and the NC groups.

As shown in Figure 6B-6D, the expression of p-PI3K in GSCs-U87, GSCs-U251 and GSCs-GTD in the agomir-29a+pIRES2-QKI-6 groups was higher than that in the agomir-29a groups, but did not differ from that in the NC groups. The expression of p-PI3K was significantly lower in the agomir-29a+pGPU6-shQKI-6 groups and the agomir-29a groups than in the NC groups (all *P*<0.05). Furthermore, p-PI3K expression was obviously lower in the agomir-29a+pGPU6-shQKI-6 groups than in the agomir-29a groups (all *P*<0.05). There were no significant differences between the control groups and NC groups. The analyses of the AKT and ERK pathways revealed similar changes in the GSCs.

## DISCUSSION

Since the miR-29 family members share the same genomic sequence, their expressed sequences and functions are nearly the same, we focus on the function of miR-29a. Xu et al. reported that downregulation of miR-29a suppressed the malignant behavior of the U87 cell line [19]; however, the expression and function of miR-29a specifically in GSCs has remained unclear. Because GSCs promote glioblastoma recurrence and reduce the effectiveness of treatments, we conducted our experiments in this subpopulation of cells. We found that miR-29a expression was lower in GSCs-U87, GSCs-U251 and GSCs-GTD than in their respective non-GSCs (Supplementary Figure 2). We also identified the effects of miR-29a on the biological behaviors of GSCs, as we demonstrated that miR-29a overexpression in GSCs inhibited cell proliferation, migration and invasion but promoted apoptosis. These data suggested that miR-29a is a tumor suppressor in GSCs, and that downregulation of miR-29a in GSCs may promote glioblastoma development (Supplementary Figure 3 and 4). Consistent with our results, previous studies have indicated that overexpression of miR-29a promotes GSC apoptosis by inhibiting MCL1 expression [20]. MiR-29a was also reported to suppress the growth and invasion of gastric cancer cells by downregulating *VEGF-A* [21]. Further, miR-29a was found to be downregulated in hepatocellular carcinoma, and was shown to inhibit cell proliferation by downregulating *SPARC* [22]. However, miR-29a was found to be upregulated in breast cancer [8] and thus act as an oncogene. Therefore, the function of miR-29a in cancer varies, and may depend on the cellular context. In our study, all the above results demonstrated that miR-29a acts as a tumor suppressor to inhibit the malignant behaviors of GSCs.

Our data revealed that QKI-6 was localized mainly in the nucleus and partly in the cytoplasm. The positive ratio of QKI-6 staining tended to increase from WHO glioma grades I to IV. QKI-6 expression was greater in GSCs-U87, GSCs-U251 and GSCs-GTD than in their respective non-GSCs. Furthermore, QKI-6 inhibition reduced cell proliferation, migration and invasion but promoted apoptosis, whereas *QKI-6* overexpression had the opposite results in GSCs. All these results suggested that in GSCs, overexpression of QKI-6 could promote oncogenic transformation.

Li et al. demonstrated that in human brain tissue, QKI-7 was expressed more strongly than QKI-6, whereas QKI-5 was scarcely expressed. They also found that QKI isoform expression was altered in approximately 30% (6/20) of human glioblastomas, but was unchanged in all the schwannomas and meningiomas tested. Among the 20 glioblastoma samples, QKI-7 was not detected in five specimens, QKI-6 was not detected in two specimens, and QKI-5 was not detected in three specimens; however, all three QKI isoforms were detected in all schwannoma and meningioma samples [23]. While the above data suggested that QKI-7 was downregulated in some of the glial tumors, the expression of QKI-6 and QKI-5 in glial tumors was still unclear. Another finding by Pilotte et al showed that only QKI-7 was found to induce apoptosis *in vitro*, while QKI-5 and QKI-6 were found to suppress apoptosis by forming heterodimers with QKI-7 [24]. It will be important to evaluate the different QKI isoform abnormalities in glioma samples on a larger scale, and to elucidate their precise functions in tumorigenesis in future studies.

In this study, *QKI-6* was predicted to be the direct target of miR-29a, and this prediction was confirmed by a dual-luciferase reporter assay. Additionally, the overexpression of miR-29a was found to inhibit QKI-6 expression. MiR-29a might downregulate gene expression by inhibiting translation through its complementary binding to the 3′-UTR of the target *QKI-6* mRNA (Supplementary Figure 5). We further investigated whether QKI-6 was involved in the tumor suppressor activity of miR-29a. Our data indicated that simultaneously upregulating the tumor suppressor gene miR-29a and downregulating the oncogene QKI-6 reduced the proliferation, invasion and migration of GSCs and increased their apoptosis, even more so than overexpressing miR-29a alone. Overexpression of *QKI-6* reversed the inhibitory effects of miR-29a overexpression on GSCs, whereas QKI-6 inhibition enhanced those effects (Supplementary Figure 6). Our *in vivo* studies also confirmed that miR-29a overexpression, combined with QKI-6 knockdown, resulted in the smallest tumors and the highest survival rate in nude mice. Thus, both miR-29a and QKI-6 might be promising targets for glioma therapy. All the above results illustrated that miR-29a acts as a tumor suppressor in GSCs and can inhibit the oncogenic behaviors of GSCs by downregulating *QKI-6*.

Previous studies have demonstrated that WTAP is overexpressed in glioblastoma tissues and promotes cell migration and invasion [18]. Our results indicated that WTAP is localized throughout the nucleoplasm and is overexpressed in glioma tissues and GSCs, suggesting that it is an oncogene in glioma and GSCs. Supporting our results, other studies have revealed that WTAP promotes tumor cell proliferation in hepatocellular carcinoma [25], and increases the migration and invasion of cholangiocarcinoma cells by upregulating *MMP7* [26]. We also predicted that *WTAP* is a target gene of QKI-6, because *WTAP* contains the QRE motif in its 3′-UTR. Consistent with our prediction, inhibition of QKI-6 reduced the expression of WTAP, whereas *QKI-6* overexpression increased WTAP expression. When a *QKI-6* construct without the *WTAP* binding site was overexpressed, WTAP protein levels did not change. Furthermore, overexpression of miR-29a inhibited the expression of WTAP by downregulating QKI-6, whereas overexpression of QKI-6 combined with miR-29a reversed the reduction in WTAP expression. The above data indicated that *WTAP* is a direct target of QKI-6 and thus participates in the tumor suppressive effects of miR-29a in GSCs.

WTAP was found to promote the migration and invasion of glioblastoma cells by stimulating the epidermal growth factor receptor (EGFR). About half of the glioblastomas exhibit EGFR anomalies, including amplification and mutation of the *EGFR* gene and/or increased EGFR protein expression [27]. Downstream signaling pathways of EGFR include the PI3K/AKT and mitogen-activated protein kinase pathways [28]. In addition, a previous study indicated that EGFR phosphorylation activates the downstream PI3K/AKT pathway [29]. Mitogen-activated protein kinases are a superfamily of protein serine–threonine kinases including ERKs. Therefore, we examined the PI3K/AKT and ERK signaling pathways to clarify the molecular pathways whereby miR-29a-induced downregulation of WTAP suppresses oncogenic behavior in GSCs. Our data suggested that overexpression of miR-29a inhibits the expression of WTAP and the activation of the PI3K/AKT and ERK pathways by downregulating QKI-6. Thus, reduced expression of WTAP blocks the activation of PI3K/AKT and ERK signaling pathways, which in turn inhibits the proliferation, migration and invasion of GSCs and promotes their apoptosis. Consistent with our conclusion, Lin et al. reported that inhibition of histamine receptor 3 suppressed glioblastoma invasion by inactivating the PI3K/AKT and MEK/ERK pathways in gliomas [30]. MiR-203 was found to inhibit glioma cell migration by disrupting the ROBO1/ERK/MMP-9 signaling axis [31].

This study is the first demonstration that miR-29a inhibits the proliferation, migration and invasion of GSCs but promotes their apoptosis by directly inhibiting *QKI-6*. *QKI-6* was found to be oncogenic in GSCs. Thus, inhibition of QKI-6 reduced WTAP expression and further blocked the activation of the PI3K/AKT and ERK signaling pathways. In conclusion, we have presented a novel characterization of miR-29a in GSCs, which suggests that miR-29a agomir therapy may be a promising treatment for human glioblastoma.

## MATERIALS AND METHODS

### Cell culture

Human glioblastoma cell lines U87 (passage 6-12) and U251 (passage 8-15), along with human embryonic kidney (HEK) 293T cells (passage 8-15), were purchased from the Shanghai Institutes for Biological Sciences Cell Resource Centre. U87 and 293T cells were grown in Dulbecco's Modified Eagle Medium (DMEM) with high glucose and 10% fetal bovine serum (FBS, Gibco, Carlsbad, CA, USA). U251 cells were grown in DMEM/F-12 medium with 10% fetal bovine serum (Gibco). All cells were incubated in humidified air at 37 °C with 5% CO_2_.

### Patient specimens

Glioblastoma tissues were obtained from patients undergoing surgery at the Department of Neurosurgery, Shengjing Hospital of China Medical University. Informed consent was obtained from all five patients (four males and one female, ranging from 41 to 65 years old), and the study was approved by the Ethics Committee of Shengjing Hospital of China Medical University. The original clinical data were collected from hospital medical records. The tumor locations were: frontal lobe (2), parietal lobe (1), temporal lobe (2). None of the patients had received radiotherapy or chemotherapy prior to surgery.

### Isolation and identification of GSCs

The GSCs isolated from the U87 and U251 cell lines were named GSCs-U87 and GSCs-U251, while the GSCs isolated from glioblastoma tissues were named GSCs-GTD (glioblastoma tissue-derived). The tissues or cells were washed, dissociated and subjected to enzymatic dissociation. CD133-positive cells were separated by magnetic cell sorting with a CD133 Cell Isolation Kit. The purity of the CD133-positive cells was evaluated by flow cytometry. CD133-positive cells were maintained in DMEM/F-12 medium supplemented with basic fibroblast growth factor (bFGF, 20 ng/mL, Life Technologies Corporation, Carlsbad, CA, USA), epidermal growth factor (EGF, 20 ng/mL, Life Technologies Corporation, Gaithersburg, MD, USA) and 2% B27 (Life Technologies Corporation, Grand Island, NY, USA) without serum. The GSCs were suspended and incubated in a 25 cm^2^ non-treated cell culture flask. The CD133-positive cells are referred to as GSCs in this paper, while the CD133-negative cells are referred to as non-GSCs. Our prior published studies have demonstrated the ability of GSCs to self-renew and differentiate [32, 33].

### Tissue microarray and IHC

Tissue microarray chips were purchased. The 60 samples included three normal brain tissues, along with three grade I, nine grade II, nine grade III, two grade II-III and thirty-four grade IV astrocytomas. All 60 samples were stained with hematoxylin and eosin (H&E). Immunohistochemistry was performed with standard methodology. The tissue microarray chips were deparaffinized in xylene (100%), re-hydrated in a graded ethanol series, and then heated in citrate buffer (pH 6.0) for 30 min at 93 °C for antigen retrieval. The microarray chips were incubated with an anti-QKI-6 antibody (1:200; Abcam, UK) or anti-WTAP antibody (1:200; Pierce antibody, USA) at 4 °C overnight. On the second day, the microarray chips were washed and then incubated with the respective secondary antibodies for 30 min at room temperature. Finally, for the color reaction, the microarray chips were incubated in a 3,3′-diaminobenzidine solution for up to 10 min with occasional inspection for color development with reference to the positive control sections. The stained microarray chips were reviewed and scored independently by two experienced pathologists who were blinded to the diagnoses and clinical assessments.

Immunostained microarray chips were scored for the percentage of stained cells (P, where 0: <10%; 1: 10-49%; 2: 50-89%; and 3: >90%) and staining intensity (*I*, where 0: negative; 1: weakly positive; 2: moderately-positive; and 3: strongly positive). Based on the value of ΣPI, the stained sections were defined as having low expression [0 (−) to 1 (+)] or high expression [2 (++) to 3 (+++)].

### RNA isolation and quantitative real-time PCR

Trizol reagent (Life Technologies Corporation, Carlsbad, CA, USA) was used to isolate total RNA from GSCs and non-GSCs, and a Nanodrop Spectrophotometer (ND100) was used to evaluate the RNA concentration and quality of each sample. The total RNA was then reverse-transcribed into cDNA with a Taqman MicroRNA Reverse Transcription Kit. Real-time PCR amplification was carried out using Taqman Universal Master Mix II with the TaqMan MicroRNA Assay of miR-29a and U6 on a 7500 Fast Real-Time PCR System (Applied Biosystems, Foster City, CA, USA). U6 was used as an endogenous control. Fold-changes were calculated through the relative quantification (2^−ΔΔCt^) method.

### Cell transfection of miRNA

The miR-29a agomir (agomir-29a) and negative control (agomir-29a-NC) were synthesized by GenePharma (Shanghai, China), and were transfected into GSCs with Lipofectamine 3000 (LifeTechnologies Corporation, Carlsbad, CA, USA). The transfection efficacy was evaluated by quantitative real-time PCR, and 48 hours post-transfection was selected as the harvesting time for subsequent experiments, as the highest transfection efficacy was attained at this time point.

### Transfection and generation of stable cell lines

For the QKI-6 knockdown experiment, we transfected GSCs with plasmids containing a short hairpin RNA directed against the human *QKI-6* gene (pGPU6-shQKI-6) or the negative control (pGPU6-shQKI-6-NC) (GenePharma). In other experiments, the *QKI-6* gene or a mutant *QKI-6* gene lacking the *WTAP* binding site (V157E) was cloned into the pIRES2-EGFP plasmid vector by SalI to allow overexpression of *QKI-6* (pIRES2-QKI-6) or its mutant (pIRES2-QKI-6-Mut) (GenScript, Nanjing, China). The same negative control (pIRES2-QKI-6-NC) was used for pIRES2-QKI-6 and pIRES2-QKI-6-Mut. Stable transfection was performed with Lipofectamine 3000 and P3000 (Life Technologies Corporation, Carlsbad, CA, USA) per the manufacturer's instructions in 24-well plates with cells at about 80% confluency. The stably transfected cells were selected with culture medium containing 0.5 mg/mL G418 (Sigma-Aldrich, St Louis, MO, USA). G418-resistant cell clones were established after approximately four weeks. The transfection efficacy was determined by Western blot analysis.

### Cell proliferation assay

Cell proliferation was assessed with a Cell Counting Kit-8 (CCK8, Beyotime, Nantong, China). The transfected GSC spheres were harvested by mechanical centrifugation, trypsinized into single cells, suspended in 100 μL GSC medium, and seeded in 96-well plates at a density of 3×10^3^ cells per well. Five replicate wells were used for each group. Three 96-well plates were incubated for 24, 48, and 72 hours, respectively; then, 10 μL CCK-8 was added to each well, and the plates were incubated for another 3 hours. The absorbance at 450 nm was measured on an ELX-800 spectrometer.

### Quantitation of apoptosis by flow cytometry

Apoptosis was assessed with the Annexin V-FITC/PI kit (DOJINDO, Beijing, China) with no light for transiently transfected cells, and with the Annexin V-PE/7-AAD kit (KeyGEN BioTECH, Nanjing, China) with green fluorescence for stably transfected cells. Cells were harvested and stained according to the manufacturer's instructions, and then were analyzed by flow cytometry. Data analysis was performed with CELLQuest 3.0 software. The upper right quadrant represents late apoptosis, while the lower right quadrant represents early apoptosis. The calculation of the total apoptotic rate included both the upper and lower right quadrants.

### Cell migration and invasion assay

For the cell migration and invasion assay, a 24-well chamber with 8-μm pores was used. First, 1×10^5^ cells were seeded in GSC medium in the upper chamber. Then, the lower chamber was loaded with 600 μL of medium containing 10% FBS. After incubation at 37 °C for 48 hours, the cells in the upper chamber were removed carefully with a cotton swab. The cells that had traversed the membrane were fixed and then stained with 20% Giemsa. For the cell invasion assay, the upper chamber was pre-coated with a 500 ng/μL Matrigel solution (BD, Franklin Lakes, NJ, USA) and seeded with 1×10^5^ cells, with the remainder of the procedure being similar to the migration assay. All chambers were photographed, and five randomly-selected fields were counted under a microscope.

### Western blot analysis

Western blotting was performed by standard methods. The PVDF membranes were incubated overnight at 4 °C with primary antibodies as follows: anti-QKI-6 (1:750, Millipore Corporation, CA, USA), anti-WTAP (1:3000, Proteintech Group, Chicago, IL, USA), anti-PI3K, anti-p-PI3K, anti-AKT, anti-p-AKT, anti-ERK1/2, anti-p-ERK1/2 (1:500, Santa Cruz Biotechnology), and anti-GAPDH (1:1000, Santa Cruz Biotechnology). The membranes were washed and then incubated with a horseradish peroxidase-conjugated secondary antibody (1:5000, Proteintech Group) at room temperature for two hours. Finally, signals were detected with an enhanced chemiluminescence detection system. The relative integrated density values (IDVs) were measured with FluorChem 2.0 software and calculated with GAPDH as the internal control.

### Reporter vector constructs and luciferase assays

We used bioinformatics databases (Starbase v2.0, miRcode and RNAhybrid) to search for the miR-29a binding site in *QKI-6*. The 3′-UTR fragment of the wild-type *QKI-6* gene (*QKI-6*-3′-UTR-Wt) or the mutant gene with respect to the predicted miR-29a binding sites (*QKI-6*-3′-UTR-Mut) were subcloned into pmirGLO Dual-luciferase miRNA Target Expression Vectors (GenePharma). HEK 293T cells were seeded in a 24-well plate and co-transfected with *QKI-6*-3′-UTR-Wt (or *QKI-6*-3′-UTR-Mut) and agomir-29a by means of Lipofectamine 3000. After the cells were incubated for 48 hours at 37 °C, the luciferase reporter assay was performed with the Dual-Luciferase Reporter Assay System (Promega, Madison, WI, USA).

### Animal studies

For the *in vivo* xenograft assays, four-week old BALB/C athymic nude mice were purchased from the Shanghai Laboratory Animal Center and raised in laminar flow cabinets under specific pathogen-free conditions. The nude mice were divided into four groups according to the injection, as follows: control group (only GSCs), miR-29a (+) group (GSCs stably overexpressing miR-29a), pGPU6-shQKI-6 group (GSCs stably overexpressing the QKI-6 inhibition plasmid), and miR-29a (+) + pGPU6-shQKI-6 group (GSCs stably overexpressing miR-29a and the QKI-6 inhibition plasmid). Each group contained 15 mice. Mice were injected subcutaneously with 3×10^5^ cells in the right axillary fossa. Tumor size was measured every five days until 60 days after injection, and was calculated by the formula: volume (mm^3^) = length×width^2^/2. For survival analysis in orthotopic inoculations, 3×10^5^ cells were stereotactically implanted into the right striatum of nude mice. The number of surviving nude mice was recorded until 60 days post-injection, and survival was analyzed with the Kaplan–Meier survival curve. All the above procedures followed protocols approved by the Animal Care Committee of China Medical University.

### Statistical analysis

All statistical analyses were carried out with SPSS 17.0 statistical software, and the data are expressed as means ± standard deviations (SDs). Statistical significance was calculated by Student's *t*-test or one-way ANOVA. Statistical significance was set at *P*<0.05.

## SUPPLEMENTARY MATERIALS FIGURES


