# Supplementary material for: Overexpression of miR-29a reduces the oncogenic properties of glioblastoma stem cells by downregulating Quaking gene isoform 6
Source: Oncotarget. 2017 Feb 15;8(15):24949–63. doi: 10.18632/oncotarget.15327 (PMC5421901; doi:10.18632/oncotarget.15327)
Supplement: Supplementary file 1 [file oncotarget-08-24949-s001.pdf]

Overexpression of miR-29a reduces the oncogenic properties of glioblastoma stem cells by downregulating Quaking gene isoform 6

SUPPLEMENTARY FIGURES

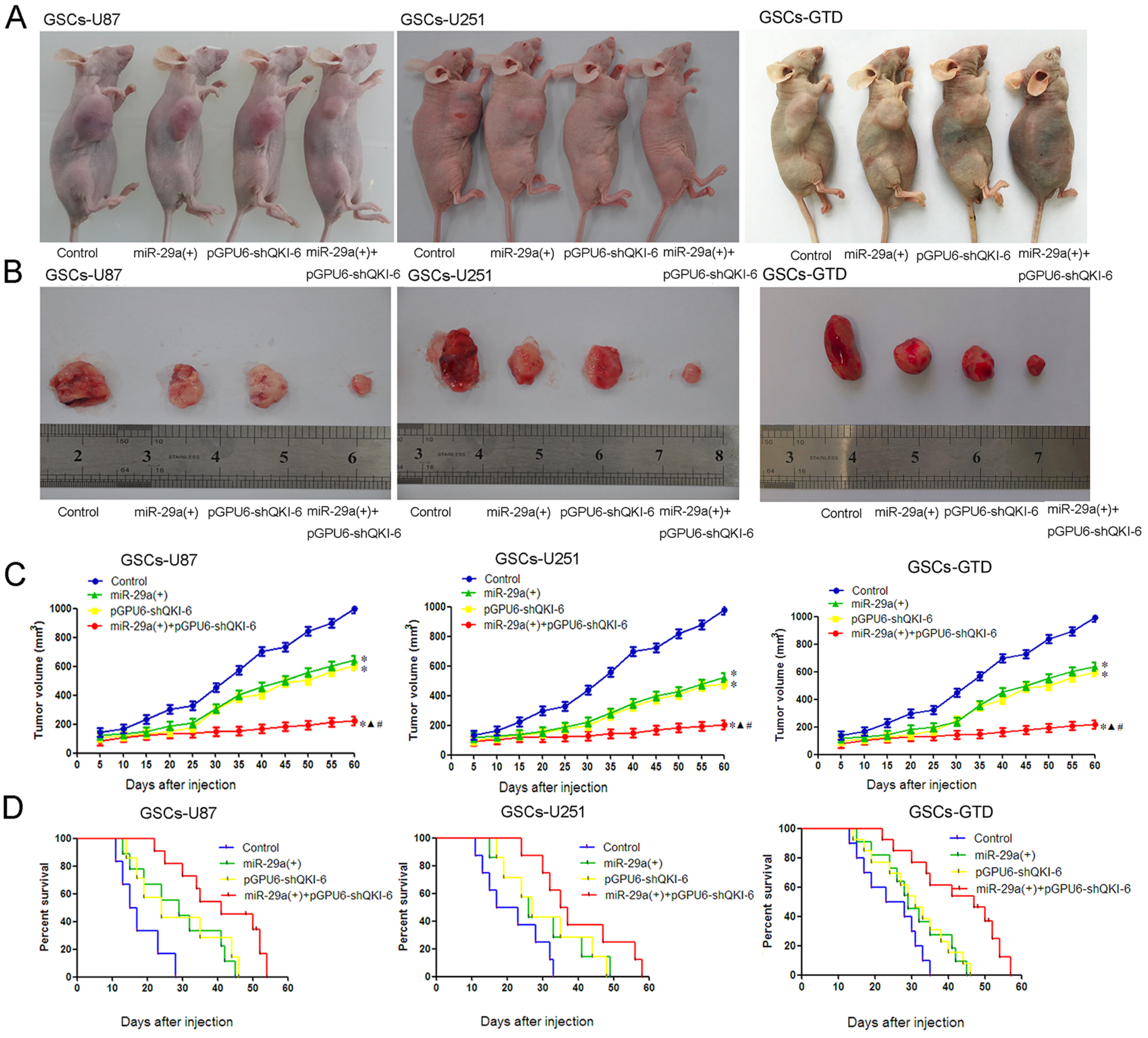

Supplementary Figure 1: *In vivo* tumor xenograft study. A. Nude mice carrying tumors from the respective groups are shown. **B.** A sample tumor from a representative group is shown. **C.** Tumor growth curves in nude mice. Mice were subcutaneously injected with 3×10^5 cells in the right axillary fossa. The tumor volume was calculated every five days after injection. $^{*}P<0.05$ vs. control group, $^{\#}P<0.05$ vs. miR-29a (+) group, $^{\blacktriangle}P<0.05$ vs. pGPU6-shQKI-6 group. **D.** The survival curves for 60 days (n=15). Nude mice were stereotactically implanted with 3×10^5 cells in the right striatum.

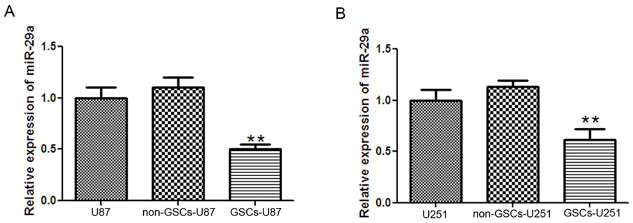

Supplementary Figure 2: The expression of miR-29a in glioblastoma cell lines, non-GSCs and GSCs. A. MiR-29a expression was significantly lower in GSCs-U87 than in non-GSCs, but did not differ between U87 cells and non-GSCs. $^{**}P$<0.01 vs. non-GSCs-U87. **B.** The expression of miR-29a in GSCs-U251, non-GSCs-U251 and U251 cells. $^{**}P$<0.01 vs. non-GSCs-U251.

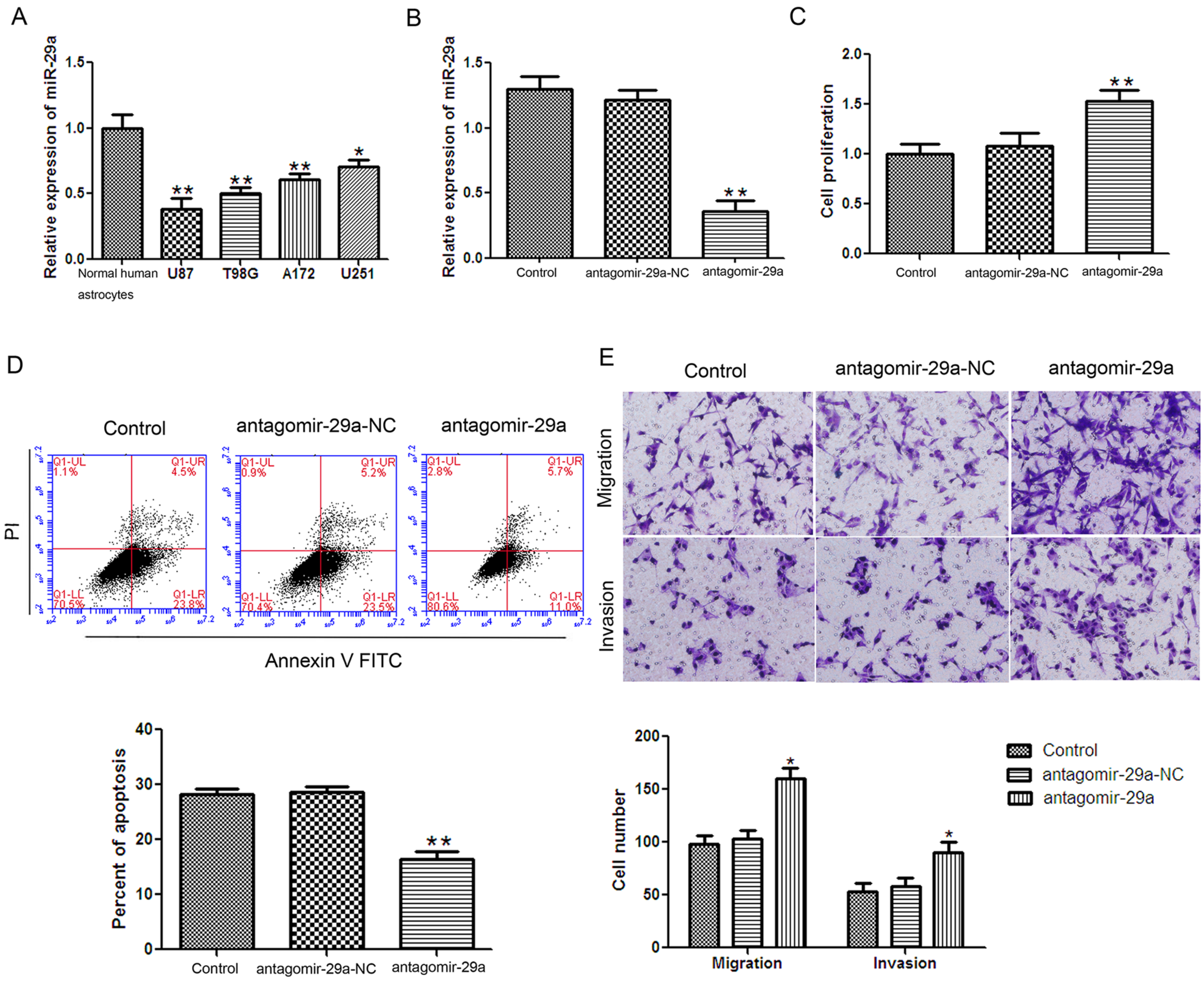

Supplementary Figure 3: Anti-miR-29a reversed the cancer suppressive effects of miR-29a in U251 cells. A. U251 cells expressed the highest endogenous levels of miR-29a among the U87, T98G, A172 and U251 glioblastoma cell lines. $^{*}P$<0.05, $^{**}P$<0.01 vs. normal human astrocytes. **B.** The transfection efficiency of antagomir-29a (anti-miR-29a). $^{**}P$<0.01 vs. antagomir-29a-NC group. **C.** Anti-miR-29a promoted the proliferation of U251 cells. **D.** Flow cytometry analysis of U251 cells treated with anti-miR-29a. **E.** Anti-miR-29a reversed the inhibitory effects of miR-29a on U251 migration and invasion. Representative images and accompanying statistical plots are presented. Values represent the mean ± SD from five independent experiments. $^{*}P$<0.05, $^{**}P$<0.01 vs. antagomir-29a-NC group. Scale bar represents 80 µm. The photographs were taken at 200× magnification.

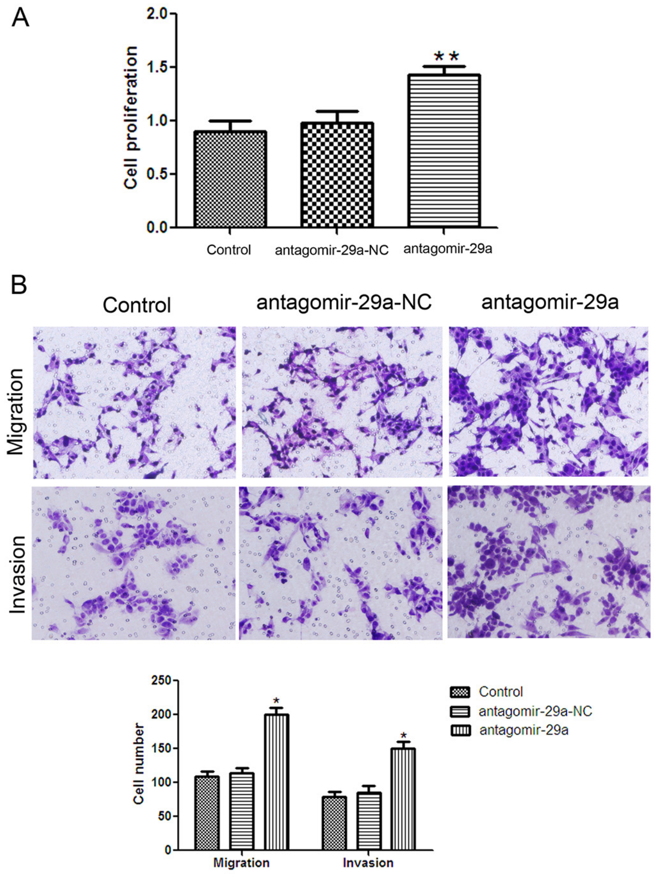

Supplementary Figure 4: anti-miR-29a promotes cell proliferation, migration and invasion in non-GSCs-U251. A. CCK8 assay to evaluate the effect of anti-miR-29a on non-GSCs-U251 proliferation **B.** Quantification of cell migration and invasion in non-GSCs-U251 downregulating miR-29a. Representative images and accompanying statistical plots are presented.$^{*}P<0.05$, $^{**}P<0.01$ vs. antagomir-29a-NC group. Scale bar represents 80 μm. The photographs were taken at 200× magnification.

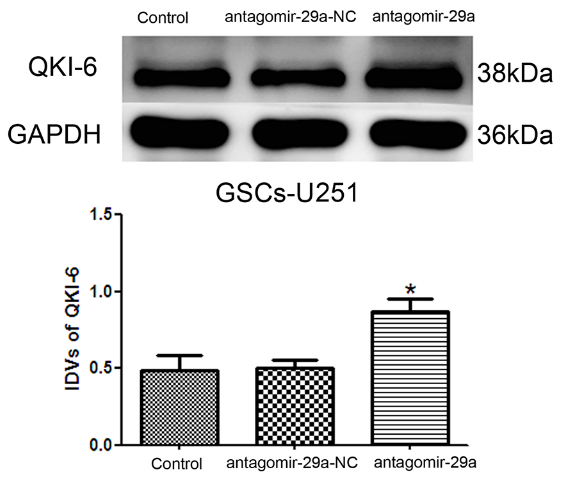

Supplementary Figure 5: Western blot results demonstrated that the protein levels of QKI-6 were significantly greater in anti-miR-29a GSCs-U251 than in anti-miR-29a-NC GSCs-U251. GAPDH was used as an internal loading control. Accompanying graphs display the densitometry analysis of protein expression. Values represent the mean ± SD from five independent experiments.$^{*}P<0.05$ vs. anti-miR-29a-NC group. IDVs represent the relative integrated density values.

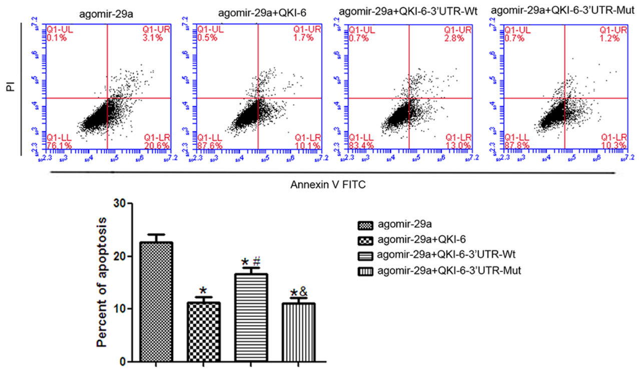

Supplementary Figure 6: Mir-29a induces apoptosis via regulating QKI6. GSCs-U87 was transfected with agomir-29a together with QKI-6 without 3'UTR (agomir-29a+QKI-6), agomir-29a together with QKI-6 without mir-29a binding site (agomir-29a+QKI-6-3'UTR-Mut), the apoptosis still reduced. $^{*}P<0.05$ vs. agomir-29a group, $^{\#}P<0.05$ vs. agomir-29a+QKI-6 group, $^{\&}P<0.05$ vs. agomir-29a+QKI-6-3'UTR-Wt group.
